# Mercury from Fish Does Not Reduce Children’s IQs

**DOI:** 10.1289/ehp.114-a399c

**Published:** 2006-07

**Authors:** Joel Schwartz

**Affiliations:** American Enterprise Institute, Sacramento, California, E-mail: joel@joelschwartz.com

Trasande et al. (2005) concluded that pre-natal methylmercury (MeHg) exposure is reducing children’s IQs (intelligence quotients), costing $8.7 billion/year. They achieved this high estimate *a*) by assuming that IQ reductions occur at MeHg exposures near or even below the 5.8 μg/L reference dose (RfD), although there is no evidence for IQ reductions even at much higher exposures; and *b*) by overstating by nearly a factor of three the fraction of newborns with MeHg exceeding the RfD. I believe that their analysis is flawed, invalid, and not appropriate as an input to policy decisions.

Trasande et al. (2005) assumed that 10% of newborns are exposed prenatally to MeHg exceeding the RfD. However, the appropriate value is 3.6%. Trasande et al. made two errors. First, they used a lower RfD than 5.8 μg/L, based on the observed enrichment of MeHg in umbilical cord blood relative to maternal blood. However, the current RfD already accounts explicitly for this enrichment through an uncertainty factor of 3.15 applied to the benchmark dose lower limit [U.S. Environmental Protection Agency (EPA) 2001]. Second, they assumed that women 16–49 years of age measured during 1999–2000 accurately represented MeHg levels in pregnant women (Mahaffey et al. 2004). National Health and Nutrition Examination Survey (NHANES) data collected during 1999–2002 (Jones et al. 2004), available before Trasande et al. (2005) submitted their manuscript, show the 95th percentile MeHg level for pregnant women to be 32% below Trasande et al.’s value.

If any MeHg exposure above the RfD reduced IQ, there would still be cause for concern. However, there is no evidence for IQ reductions even at exposures several times the RfD.

Previous studies in the Seychelles Islands (Myers et al. 2003) and New Zealand (Crump et al. 1998) did not find IQ reductions at any MeHg exposure. A study in the Faroe Islands (Grandjean et al. 1999) did not measure IQ. Many children in these studies had prenatal MeHg exposures exceeding 10 times the RfD. The claim of IQ reductions in Americans is even weaker because Americans’ MeHg exposures are far lower. Of 629 pregnant women measured by NHANES, the highest exposure was 3.7 times the RfD (Centers for Disease Control and Prevention 2005). Among those exceeding the RfD, 75% were below twice the RfD.

Trasande et al. (2005) cited results from the Faroe Islands (Grandjean et al. 1999) to claim IQ reductions, but this study is less compelling than the Seychelles study (Myers et al. 2003b) for assessing Americans’ risks: *a*) the Seychellois are exposed to MeHg through ocean fish, similar to Americans, whereas the Faroese are exposed through whale meat (Myers et al. 2003b); *b*) the Seychellois are ethnically diverse, but the Faroese are homogeneously Scandinavian (Rice et al. 2003); and *c*) the Seychelles study used hair MeHg to measure exposure, and the Faroes study used cord blood. Hair MeHg has been calibrated with fetal brain levels, but cord blood has not (Cernichiari et al. 1995; Myers et al. 2003a).

Despite the advantages of the Seychelles study, Trasande et al. (2005) dismissed it, claiming that the National Research Council (NRC 2000) “opined that the most credible of the three prospective epidemiologic studies was the Faroe Islands investigation.” In reality, referring to all three studies, the NRC (2000) concluded that “each of these studies was well designed and carefully conducted.” Nevertheless, the NRC “concluded that a well-designed study with positive effects provides the most appropriate public-health basis for the RfD.” The NRC thus excluded the Seychelles study not because of the quality of the study but because the study found that MeHg did not cause any harm.

Trasande et al. (2005) also made other errors:

- They claimed that the New Zealand study reported IQ reductions, citing Kjellstrom et al. (1986, 1989). However, they omitted Crump et al.’s (1998) reanalysis, coauthored with Kjellstrom, which superseded previous reports and found no IQ reduction.
- They claimed that the Seychelles study had only half the statistical power of the Faroes study. The studies actually have similar power (Myers et al. 2003; NRC 2000).
- They claimd the NRC concluded that MeHg reduces IQs even at exposures lower than the RfD. However, the NAS cautioned that the cohort studies were incapable of assessing effects of exposures near the RfD, because hardly any children had such low MeHg exposures (NRC 2000).

The weight of the evidence indicates that MeHg, even at exposures substantially greater than the highest U.S. levels, does not reduce children’s IQ. The evidence against IQ reductions is particularly strong for MeHg exposures from fish.

Trasande et al. (2005) relied on mistaken assumptions regarding exposures to and effects of MeHg, and misinterpreted or omitted contrary evidence. Therefore, I consider their analysis to be fundamentally flawed and invalid.
